# Intensity-modulated ventricular irradiation for intracranial germ-cell tumors: Survival analysis and impact of salvage re-irradiation

**DOI:** 10.1371/journal.pone.0226350

**Published:** 2019-12-20

**Authors:** Ana Carolina Pires de Rezende, Eduardo Weltman, Michael Jenwei Chen, Juliana Karassawa Helito, Ícaro Thiago de Carvalho, Roberto Kenji Sakuraba, Nasjla Saba Silva, Andrea Maria Cappellano, Nelson Hamerschlak

**Affiliations:** 1 Department of Radiation Oncology, Hospital Israelita Albert Einstein, São Paulo, Brazil; 2 Department of Radiation Oncology, Faculdade de Medicina da Universidade de São Paulo, São Paulo, Brazil; 3 Department of Radiation Oncology, Instituto de Oncologia Pediátrica - Grupo de Apoio ao Adolescente e à Criança com Câncer (GRAACC) da Universidade Federal de São Paulo, Sao Paulo, Brazil; 4 Department of Pediatric Oncology, Instituto de Oncologia Pediátrica - Grupo de Apoio ao Adolescente e à Criança com Câncer (GRAACC) da Universidade Federal de São Paulo, São Paulo, Brazil; 5 Department of Hematology and Clinical Oncology, Hospital Israelita Albert Einstein, São Paulo, Brazil; University of Nebraska Medical Center, UNITED STATES

## Abstract

**Background and purpose:**

The roles of surgery, chemotherapy, and parameters of radiation therapy for treating very rare central nervous system germ cell tumors (CNS-GCT) are still under discussion. We aimed to evaluate the survival and recurrence patterns of patients with CNS-GCT treated with chemotherapy followed by whole ventricle irradiation with intensity-modulated radiation therapy.

**Materials and methods:**

We reviewed the clinical outcomes of 20 consecutive patients with CNS-GCT treated with chemotherapy and intensity-modulated radiation therapy from 2004 to 2014 in two partner institutions.

**Results:**

Twenty children with a median age of 12 years were included (16 males). Sixteen tumors were pure germinomas, and 4 were non-germinomatous germ cell tumors (NGGCT). All patients were treated with intensity-modulated radiation therapy guided by daily images, and 70% with volumetric intensity-modulated arc radiotherapy additionally. The median dose for the whole-ventricle was 25.2 Gy (range: 18–30.6 Gy) and 36 Gy (range: 30–54 Gy) for the tumor bed boost. The median post-radiation therapy follow-up was 57.5 months. There were 3 recurrences (2 NGGCT and 1 germinoma that recurred as a NGGCT), with 1 death from the disease and the other 2 cases each successfully rescued with chemotherapy and craniospinal irradiation. The overall survival at 5 years was 95% and disease-free survival was 85%.

**Conclusions:**

The results of this study suggest that the combined use of chemotherapy followed by whole ventricle irradiation with intensity-modulated radiation therapy is effective for CNS-GCTs, especially pure germinomas. Even being rescued with craniospinal irradiation, the NGGCT cases have markedly worse prognoses and should be more rigorously selected for localized treatment.

## Introduction

Central nervous system germ cell tumors (CNS-GCT) are a very rare, heterogeneous group of malignancies consisting of histological subtypes with different prognostic profiles [1]. They account for 3% of pediatric brain tumors, except in Asian countries, where the incidence increases to 11% [2–4]. The World Health Organization classifies CNS-GCT into two histological subgroups with high prognostic value: pure germinomas (PG) and non-germinomatous germ cell tumors (NGGCT) [5]. PG are more common, representing around two-thirds of CNS-GCT, and have a more favorable prognosis. NGGCT include all GCT with some germinal component of malignancy and/or any tumor that secretes alpha-fetoprotein (AFP) or high levels (> 100–200 IU) of ß-human chorionic gonadotropin (ß-HCG) [6,7].

Treatment of CNS-GCT is still controversial with discussions on how to establish histology parameters, the roles of surgery, chemotherapy (CT), and radiation therapy (RT). Studies have aimed to reduce treatment intensity, either by the exclusive use of CT, or of CT combined with restricted radiation volumes covering only the tumor bed. However, the disease recurrence rates were found unacceptable, reaching 48% when RT was omitted, and the use of irradiation in very focal volumes (i.e only the tumor bed) resulted in high recurrence rates in the non-irradiated ventricular regions [8–10]. The current trend is to use neoadjuvant CT (regimens based on carboplatin and etoposide with or without iphosphamide). Patients with a good response are offered a consolidative treatment with whole ventricle irradiation followed by a tumor bed boost [11,12]. There are ongoing studies, such as the ACNS1123 of the Children’s Oncology Group—COG (NCT01602666), aiming to reduce radiation doses given to these children without compromising the high cure rates. However, preliminary results are not expected to become available until 2024.

The neurocognitive effects of cranial RT in children have been studied under various scenarios. In CNS prophylaxis for acute lymphoblast leukemia, the use of 24 Gy of radiation to the whole brain (combined with intrathecal CT with methotrexate) has been associated with a reduction of the intelligence quotient (IQ) 5 years following RT, poor scholar performance, a worsening perception of self-image, and higher rates of psychological distress [13]. Related toxicities were reduced (or not detected) when doses of 14–18 Gy were administered. In medulloblastomas, the post-RT IQs were 10–15 points better with whole brain doses of 23.5 Gy compared with 36 Gy [14]. The differences among study conclusions can be explained by the inability of some series to correlate the complex interactions between the dose, the volume, and the patient’s age, with a longer follow-up. Merchant et al. suggested that different regions of the brain, especially the supratentorial area, are important in the development of the cognitive decline associated with RT [15].

The use of technology is necessary to minimize secondary toxicities due to radiation. Compared with conventional or three-dimensional conformal radiotherapy (3D-RT) intensity-modulated radiation therapy (IMRT) is a technique capable of delivering the prescribed dose of radiation in a precisely targeted way in this case to the tumor bed and the ventricles, sparing adjacent structures and thereby reducing the amount of normal tissue that receives high-dose radiation unnecessarily [16]. This is an important consideration because studies have already shown there is a dose-dependent relationship between cerebral irradiation and the reduction of cognitive function and IQ [17,15].

Although CNS-GCT are rare, there are reports of preserved neurocognitive, social, and emotional functions in pediatric and adolescent PG patients treated with CT followed by irradiation using IMRT to deliver reduced doses to the ventricular system [18]. The objective of this study was to report survival and recurrence in patients with CNS-GCT using neoadjuvant CT followed by irradiation of the ventricular system using IMRT.

## Materials and methods

### Study design and ethics

In this retrospective study, we reviewed the medical records of all consecutive patients diagnosed between 2004 and 2014 with CNS-GCT (n = 20). These patients were treated with whole ventricle irradiation using IMRT in two institutions, collaborating through a public-private partnership, in São Paulo, Brazil. The Ethics committee of both hospitals (Hospital Israelita Albert Einstein and Grupo de Apoio ao Adolescente e à Criança com Câncer, GRAACC) where the children were treated approved the protocol of this study, which was carried out in accordance with the Code of Ethics of the World Medical Association (Declaration of Helsinki). The legal guardians provided written informed consent for treatment. Consent for inclusion in this study was waived since this was a retrospective study based on medical records, and no patient was identified.

### Patients and treatments

All patients went through the following staging procedures before starting treatment: brain and spine MRI, baseline ophthalmologic exam, CSF cytology, serum and CSF markers (ß-HCG and AFP). All cases were treated aiming for a curative goal using neoadjuvant CT and, prior to RT, 8 patients underwent surgical resection. Drug protocols are described in Table 1.

**Table 1:** Chemotherapy protocols used, according to histological type.

**Germinomas**		
Cycles 1 to 4	Carboplatin	300 mg/m^2^/day
	Etoposide	225 mg/m^2^/day
**Germ cell tumors**		
Cycles 1, 3, and 5	Carboplatin	300 mg/m^2^/day
	Etoposide	225 mg/m^2^/day
Cycles 2, 4, and 6	Cyclophosphamide	1.2 g/m^2^/day
	Etoposide	225 mg/m^2^/day

RT was delivered to the whole ventricular system, using IMRT followed by a boost dose only to the tumor bed. Two patients did not receive this boost. Before starting radiation therapy, all patients underwent a simulation procedure with a thermoplastic mask immobilization, followed by computed tomography (CT) of the area. Images generated were transferred to a computerized planning system (Eclipse—Varian Medical Systems, Palo Alto, CA) to be digitally merged with pre- and post-chemotherapy diagnostic magnetic resonance imaging (MRI).

The prescribed dose-fraction, including the tumor bed boost, varied from 1.5 to 2 Gy per day, with 5 fractions per week. The gross target volume (GTV) corresponded to the primary lesion detected in the first diagnostic MRI added to any residual disease remaining after the initial therapy, as detected in the most recent restaging MRI. In cases of total remission following CT and/or surgery, the GTV corresponded, exclusively, to the site of the primary lesion detected in the first diagnostic MRI. For the tumor bed boost, a clinical target volume (CTV) was created corresponding to the GTV with additional margins of 1.0 to 1.5 cm. For ventricular irradiation, the CTV corresponded to the whole ventricular system, with an additional volumetric margin of 0.3 to 0.5 cm added. Additional 0.3 to 0.5 cm margins were applied to the CTV for each planning target volume (PTV).

All treatments were performed with a 6 MV linear accelerator (23EX, Varian Medical Systems), using the dynamic IMRT technique (sliding window) or volumetric intensity-modulated arc radiotherapy (VMAT), all guided by daily images (IGRT). The plans were calculated using Eclipse software according to the medical prescriptions for target volumes, respecting the pre-established restrictions from the dose-volume histograms, according to Emami et al. [19]. Dosimetric quality controls were conducted using an ionization chamber and portal dosimetry. Besides receiving a technically homogeneous radiation treatment, all patients were followed by the same team of radiation oncologists and pediatric oncologists, with the same protocol of neoadjuvant CT.

### Endpoint analysis

The endpoints analyzed included overall survival, disease-free survival, recurrence patterns, and the impact of salvage RT (re-irradiation) in controlling the recurrent disease. Overall survival was defined as survival from the date of diagnosis to the date of death by any cause, excluding patients who were alive at the time of analysis. Disease-free survival was defined as survival from the date of diagnosis to the recurrence date recorded in the medical chart. The pattern of recurrences was described by histological type, radiological presentation, and changes in serum and/or fluid markers. The impact of salvage irradiation was evaluated with radiological parameters and by the normalization of tumor markers after treatment.

### Statistical analysis

Patient characteristics were described using the average, standard deviation, median, minimum, and maximum values for the quantitative variables and absolute and relative frequencies for the qualitative variables. The probabilities of overall survival and disease-free survival were estimated using the Kaplan-Meier method.

## Results

In this retrospective study, all 20 admitted patients completed their planned treatment and were thus eligible for the evaluation. The median age at the beginning of RT was 12 years old (range: 6 to 18 years), and 16 patients (80%) were male. The location of the tumors was pineal in 8 patients, suprasellar in 7, and pineal and suprasellar in 5. Sixteen patients were diagnosed with a PG (1 with a teratoma with germinomatous microfoci) and 4 with NGGCT (2 with mixed tumors). All patients had only localized intracranial disease, without evidence of cytological dissemination in the cerebrospinal fluid (CSF) or the neuraxis MRI. Six patients had positive tumor markers (ß-HCG and/or AFP) in the CSF at diagnosis.

At the end of the neoadjuvant CT, 15 patients were in complete remission from the disease. Of the 5 remaining patients with residual intracranial lesions, 2 underwent surgical resection while the other 3 had residual lesions not amenable for resection. The 20 patients were treated with whole ventricle IMRT using daily IGRT, and the VMAT technique was used in 70%. Only 2 patients did not receive a tumor bed boost. The median dose delivered to the ventricular fields was 25.2 Gy (range: 18 to 30.6 Gy) and the median dose to the tumor bed was 36 Gy (range: 30 to 54 Gy). The general characteristics of the 20 patients are summarized in Table 2.

**Table 2:** Characteristics of the patients studied.

Variable	Frequency (n = 20)
**Sex (male), n (%)**	16 (80)
**Age (years)**	
mean (SD)	11.8 (3.4)
median (min.; max.)	12 (6; 18)
**Histopathology, n (%)**	
Germinoma	16 (80)
NGGCT	4 (20)
**Serum marker, n (%)**	2 (10)
**CSF marker, n (%)**	6 (30)
**Surgery, n (%)**	7 (35)
**RT Field, n (%)**	
ventricular system + tumor bed	18 (90)
ventricular system	2 (10)
**Dose cGy (ventricles)**	
mean (SD)	2540 (370.4)
median (min.; max.)	2520 (1800; 3060)
**Dose cGy (tumor bed)**	
mean (SD)	3908.9 (786)
median (min.; max.)	3600 (3000; 5400)
**Technique, n (%)**	
VMAT	14 (70)
IMRT	6 (30)
**Recurrence, n (%)**	3 (15)

NGGCT = non-germinomatous germ cell tumors; CSF = cerebrospinal fluid; RT = radiation therapy; VMAT = volumetric intensity-modulated arc radiation therapy; IMRT = intensity-modulated radiation therapy; SD = standard deviation; min = minimum; max = maximum.

**Fig 1:**
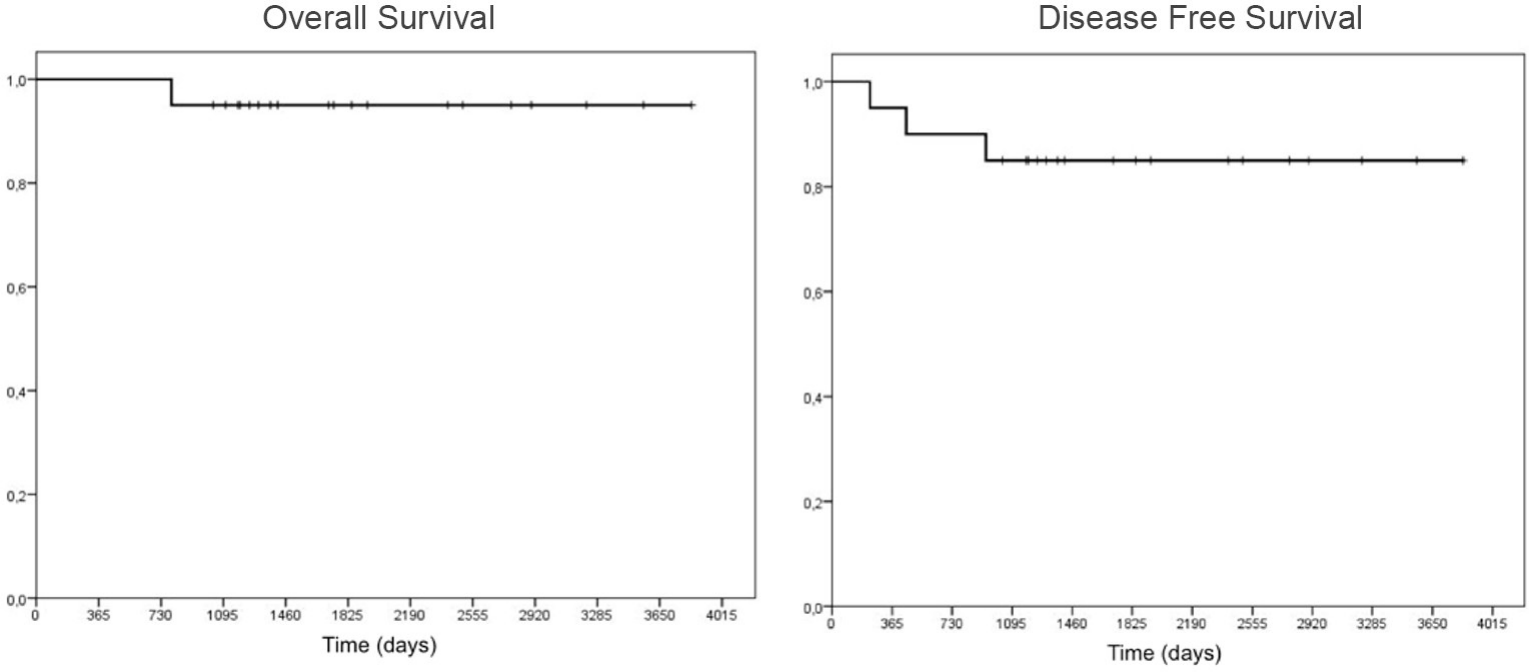
Overall survival (A) and disease-free survival (B) of patients with central nervous system germ cell tumors treated with chemotherapy followed by whole ventricle irradiation with intensity-modulated radiation therapy.

The median post-RT follow-up time was 57.5 months (range: 26.4 to 127.9 months). Overall survival was 95%, and disease-free survival was 85% (Fig 1). Considering the two different groups with markedly different prognosis, the PGs presented an overall survival of 100% and disease-free survival of 93.75%, while the NGGCT had an overall survival of 75% with disease-free survival of 50%.

**Fig 2:**
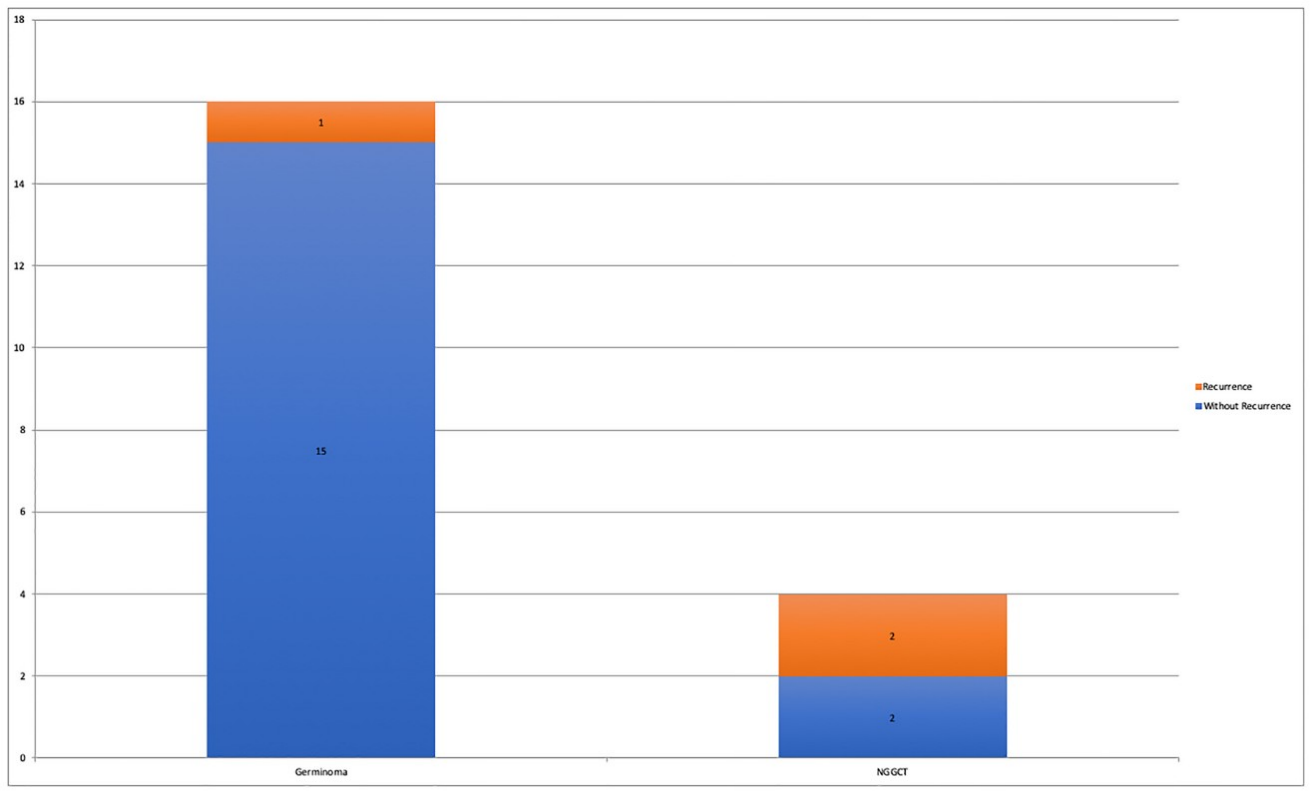
Relapses according to the histological type in patients with central nervous system germ cell tumors treated with chemotherapy followed by whole ventricle irradiation with intensity-modulated radiation therapy.

During this period, 3 recurrences were observed with only 1 death from the disease. All recurrence cases were dosimetrically reviewed, showing a good radiation dose distribution with at least 95% of the targeted volumes covered by 95% of the prescribed dose. Two patients with recurrent disease were initially NGGCT, with positive serum and CSF markers at diagnosis. The other case was originally a germinoma (with serum ß-HCG < 50 mlU/ml, negative serum AFP and negative CSF markers and cytology) that recurred as a NGGCT with positive serum and CSF markers (both ß-HCG and AFP) and CSF positive cytology. (Fig 2).

All recurrences were located in the neuraxis and were positive for CSF markers. Two of these patients also presented new lesions in the brain and spinal cord. The recurrences appeared within the first 6 months in 2 cases and only after 16 months in the third case. One of the patients with NGGCT suffered from post-CT hematological toxicity (low blood account, especially low platelets), so she received a relative final low dose for a NGGCT (36Gy instead of 50.4/55.4 Gy), presenting early recurrence in the primary tumor site and spine. Also, this patient was considered a slow responder during the initial CT treatment and was the only one in this study to die from the disease.

**Fig 3:**
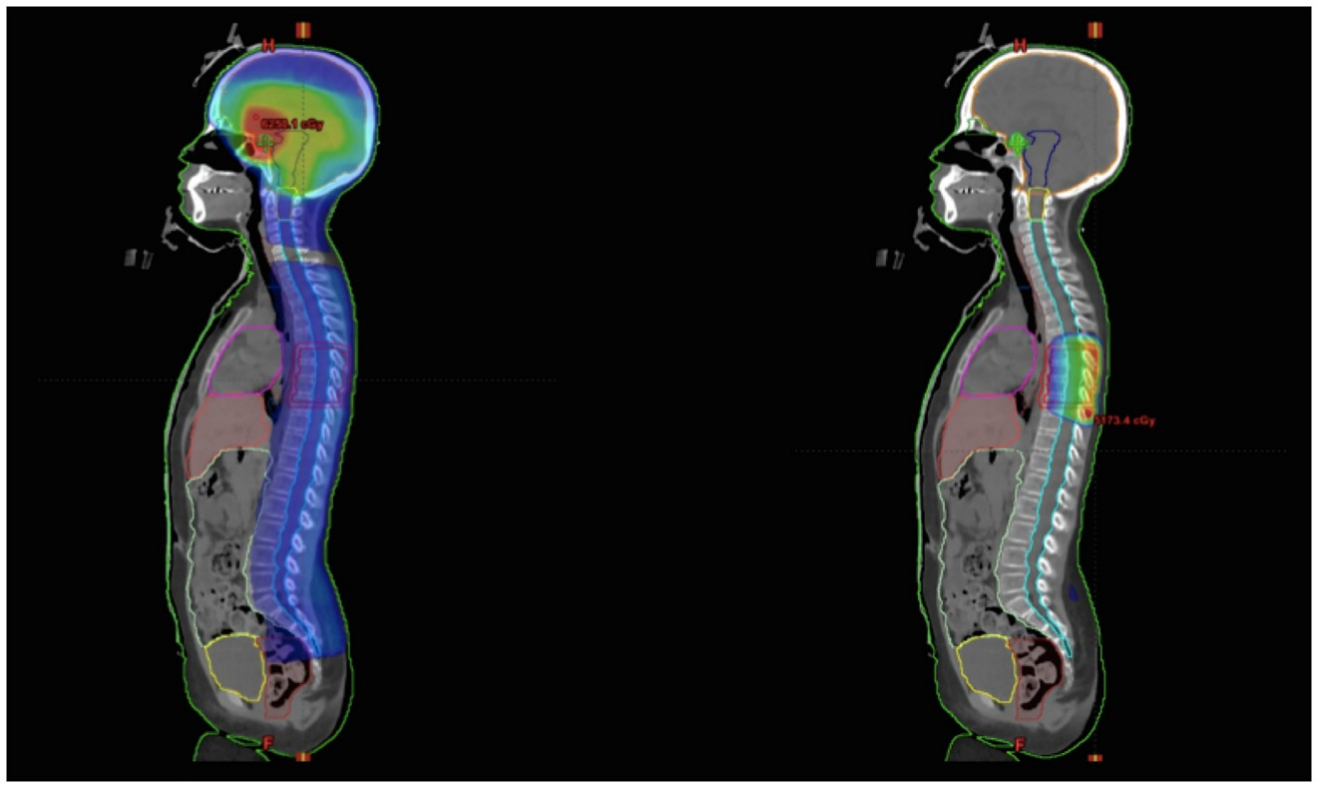
Example of re-irradiation volumes showing the distribution of the craniospinal and boost doses in the recurrence sites (blue, isodose of 25 Gy; green, of 45 Gy; and red, of 60 Gy).

When the recurrence was diagnosed, the patients received a new CT plan based on cisplatin, etoposide, and ifosfamide and 2 underwent autologous bone marrow transplants. They were then referred for RT and received craniospinal irradiation, with a boost dose to the recurrent lesions visible in the MRI. The dose distribution of one of these cases is shown in Fig 3. None of the patients presented acute or late toxicity higher than grade 1 according to the Radiation Therapy Oncology Group (RTOG) toxicity classification [20]. Two patients showed no evidence of the disease at 29 and 57 months after the last course of RT. More details on the re-irradiated patients and doses used are shown in Table 3. None of the patients were lost to follow up. S1 Table shows data from all the patients.

**Table 3:** Description of the recurrence patterns.

Histology	Serum Diagnostic Markers	CSF Diagnostic Markers	1^st^ RT (Gy)* vent/bed	Time to recurrence (since the end of 1^st^ RT)	Pattern of Recurrence	2^nd^ RT (Gy)^†^	Status (OS)
Germinoma	Negative	Low BHCG	23.4/30.6	6 months	NGGCT^‡^ CSF+ Spine Brain	CSI = 25.2 Lumbar = 30.6	Living NED (65 m)
NGGCT	AFP and ßHCG	AFP and ßHCG	25.2/36.0	6 months	CSF+ Spine Brain	CSI = 23.4 T7-8 = 36.0 CNS = 45.0	Death (28 m)
NGGCT	Negative	AFP and ßHCG	30.6/50.4	16 months	CSF+	CSI = 36.0 Cranium = 18.0	Living NED (59 m)

NGGCT = non-germinomatous germ cell tumor; AFP = alpha-fetoprotein; ßHCG = beta human chorionic gonadotropin; RT = radiotherapy; CSF+ = cerebrospinal fluid with positive markers; CSI = radiation therapy in the craniospinal axis; OS = overall; survival; NED = no evidence of disease.

*1^st^ RT(Gy) vent/bed = first course of radiation therapy with doses to the ventricles and the tumor bed, in Gy;

^†^2^nd^ RT (Gy) = second irradiation with doses in Gy;

^‡^patient initially diagnosed with germinoma that recurred as a NGGCT.

## Discussion

We report the results of the treatment of CNS-GCT pediatric patients with neoadjuvant CT, followed by whole ventricle irradiation using IMRT. To our knowledge, this is the largest Brazilian and Latin American group of patients with CNS-GCT treated using this technology. CNS-GCT are rare entities, and there is still no consensus on the best treatment approach. Although there is some evidence that isolated radiation therapy may be effective in curing these patients [21], several studies conducted over the past two decades strongly suggest the adding of CT as the first-line treatment before definitive irradiation [22]. Germinomas have a high response rate to radiation, with long-term survival rates higher than 90% using RT alone; on the other hand, NGGCT has a worse prognosis and seems to need more intensive treatment. However, the best drug protocol to be combined with irradiation and the best way to manage the use of RT in the locoregional control of the disease are still unclear, and the common late effects from craniospinal irradiation are still considered unacceptable by some experts [23].

Few randomized prospective studies have been published, and clinical decisions are often based on retrospective studies and historical data [3]. The main study (ACNS1123 COG study), which is still ongoing, intends to determine prospectively whether neoadjuvant CT followed by whole ventricle irradiation with reduced doses will succeed in maintaining the excellent historical results obtained with localized CNS-GCT treatment. In the COG study, the treatment regimen is similar to the one used in our group, with neoadjuvant CT (based on carboplatin and etoposide for PG, with the addition of ifosfamide for NGGCT) offered to the patients showing good response whole ventricle irradiation (reaching 18 Gy for PG and 30.6 Gy for NGGCT) with a tumor bed boost (reaching 30 Gy for PG and 54 Gy for NGGCT).

Other relevant studies are very heterogeneous, regarding both the number of patients and specific guidelines for each risk group. An example is one of the most robust prospective phase II studies, the SIOP CNSGCT 96: despite having a multi-institutional design involving 12 European countries, patient enrollment was difficult due to the rarity of the disease. In addition, patients were not randomized for inclusion in the treatment groups, leaving the choice of group allocation to the physician or to the institution that received them. Although there is a relatively large number of patients in Japan due to the higher incidence in Asian countries, studies carried out with Japanese patients included very distinct risk groups, with different treatment regimens and very different volumes of RT [6,8,22,24,25]. When we started treating these patients, due to the lack of prospective evidence of focal RT use versus CSI, we chose an intermediate modality using the WVI for both PG and NGGCT with localized disease, aiming to minimize the radiation dose on normal brain tissue but giving a good dose coverage to the high risk areas, the ventricles and the tumor bed [26]. Likewise, some other published studies have reported good results with local RT for this type of patient.[27–30]

Thus, even though ours is a retrospective study without a previously established dose protocol, our results are very positive, specially for the PGs, because all patients received a technically homogeneous treatment by the same team of radiation oncologists and pediatric oncologists, with the same protocol of neoadjuvant CT followed by an advanced technology RT. Additionally, considering the rarity of this disease, other important studies on CNS-GCT also included an average of 20 patients, as in our case [18,27,31–34].

Compared to Japanese, Canadian, American, and European series, we also obtained positive outcomes when we considered the two histological subtypes as distinct risk groups. For PG, we found an overall survival of 100% and disease-free survival of 93.75%, values comparable to the average values of 97–100% and 89–96%, respectively, in these international studies. More importantly, for the NGGCT a group known for its worse prognosis, we found an overall survival of 75% with disease-free survival of 50% versus 68–75% and 60–68% obtained in other comparable studies. [9,22,24,27,32].

In our patient follow-up, we observed 3 recurrences: 1 was a germinoma that recurred as non-germinomatous, and the other 2 were originally NGGCT with positive serum and CSF markers at diagnosis. Thresholds for these tumor markers are not clear-cut, leading to potential inaccuracies in diagnosis. NGGCTs are diagnosed if the AFP or HCG level are higher than certain limits defined in the CSF or serum, but some germinomas can have positive HCG in lower levels and are considered a higher risk group when compared with those that are negative for markers.[28,35]

Even with our limited sample, this recurrence pattern stands out because these two patients with recurrent disease account for 50% of all the NGGCT, and one of them, while achieving total response, received a relatively low dose for an NGGCT and was considered a slow responder to neoadjuvant CT, being the only registered death from the disease. The other children were successfully rescued with CT and a new course of radiation (CSI) with low rates of toxicity. Taken together, the results reinforce the importance of a more rigid selection in low-dose RT protocols with fields restricted to the ventricular system, especially in patients with NGGCT that may benefit from craniospinal irradiation. A Taiwanese review of 102 cases of recurrent CNS-GCT that were successfully treated showed that initial treatment with extensive volumes and higher doses of RT can complicate salvage re-irradiation. The authors pointed out that these tumors, when recurring, are highly sensitive to radiation and/or CT, similar to naïve tumors at diagnosis, and regimens combining CT with craniospinal irradiation in low doses were highly effective in obtaining sustainable control of the disease, with acceptable levels of acute and late toxicity [36].

Recently, the results of the largest prospective series of patients with intracranial malignant NGGCT, treated in the multinational European protocol SIOP-CNS-GCT-96, have reinforced the use of a more localized RT even for this group of poorer prognosis. The study showed that the combination of CT and RT for NGGCT patients, with risk-adapted RT tailored according to initial dissemination (focal for those with localized disease and CSI plus focal boost for metastatic cases), was effective at producing long-term durable treatment response [25].

On the other hand, after an interim analysis, the ACNS1123 study has closed the arm of the patients with localized NGGCTs treated with WVI prematurely, and formal reporting of the results is still awaited, showing that the optimal radiotherapy volume for localized NGGCT continues to be different globally [28]. Probably, a response-based approach would be more appropriate for the treatment of these patients.

According to QUANTEC (Quantitative Analysis of Normal Tissue Effects in the Clinic), younger age is the most important risk factor for neurocognitive decline in children who undergo cranial RT. Other risk factors include being female, the NF-1 mutation, an extension of the surgical resection, hydrocephalus, protocols including neurotoxic CT (especially high dose methotrexate), tumor location, and the irradiated brain volume [37].

The finding that IMRT is capable of significantly reducing the cerebral hemisphere volumes that receive elevated doses of radiation is relevant not only for this group of patients with a rare disease but also for pediatric patients with other CNS tumors [34,38]. Given the complex form, size, and central location of the ventricular system, the resulting PTV is usually irregular and large. In our population, the whole ventricle PTV size corresponded on average to 28% of the total brain volume [38]. Furthermore, the spared cerebral hemispheres concentrate a large amount of the external layer of brain tissue, the very region where the cerebral cortex is located.

There is a trend to consider proton beam RT as the technique of choice for the treatment of CNS tumors in children. Several studies have shown its dosimetric benefits and some are trying to demonstrate its long-term advantages [39]. Bearing in mind that the CNS-GCT is a rare group of malignancies, and most of the publications refer to other histology types, we could use for them the same rationale, knowing that Proton plans showed a consistent reduction in dose to the adjacent critical structures (for example: temporal lobes, hippocampi, cochlea and whole brain), supporting the potential for a reduction in late effects, neurocognitive development, and secondary malignancy risk in this population. [40–43].

Even with all the optimism and the expectations generated by the introduction of this new technology, recent publications about the cognitive assessments of these children did not demonstrate significant differences in performance compared with those treated with photons in modern protocols [44]. Alternatively, modern protocols with IMRT can be so successful in limiting the exposure of the surrounding healthy brain tissue that patients treated in these studies since 2002 are not experiencing the magnitude of neurocognitive decline reported in earlier studies [44]. So, there is still interest in the study of IMRT, especially in low-income countries where the proton technology is still not available, considering that in comparison to the 3D RT, IMRT can also provide a consistent dose reduction to critical structures with the potential to generate a lasting impact in these children’s lives.[38]

While we wait for the results of the ACNS1123 trial that will hopefully validate whether this approach is safe in a larger cohort of prospectively followed patients, our findings provide more information to support the use of neoadjuvant CT followed by whole ventricle irradiation and a tumor bed boost in patients with localized intracranial disease.

## Conclusions

The results of this study suggest that the combined use of chemotherapy followed by whole ventricle irradiation with IMRT is effective for CNS GCTs, specially PGs. Even being rescued with CSI, the NGGCT cases have markedly worse prognoses and should be more rigorously selected for localized treatment. Larger prospective studies might shed light on the more appropriate radiation doses and volumes for both PG and NGGCT, probably guided by a response-based approach.

## Supporting information

S1 Table Characteristics of the 20 patients. (DOCX)
