# Supplementary material for: Intensity-modulated ventricular irradiation for intracranial germ-cell tumors: Survival analysis and impact of salvage re-irradiation
Source: PLoS One. 2019 Dec 20;14(12):e0226350. doi: 10.1371/journal.pone.0226350 (PMC6924640; doi:10.1371/journal.pone.0226350)
Supplement: S1 Table — (DOCX) [file pone.0226350.s001.docx]

**S1 Table – Characteristics of the 20 patients**

| **Patient** | **Age** | **Sex** | **Histopathology** | **Serum marker** | **CSF marker** | **CSF cytology** | **Tumor Location** | **Post-CT response** | **Surgery** | **RT volumes** | **Dose Gy (ventricular system/boost)** | **RT modality** | **Relapse** | **Status** | **General complications** |
| --- | --- | --- | --- | --- | --- | --- | --- | --- | --- | --- | --- | --- | --- | --- | --- |
| 1 | 9 | M | Germinoma | Negative | Negative | Negative | Suprasellar to III ventricle | Complete | Partial (pre-CT) | Ventricular system + boost | 23.4/30.6 | VMAT | Yes | Alive, NED | Pan-hypo |
| 2 | 8 | F | Germinoma | Negative | BHCG | Negative | Suprasellar | Complete | No | Ventricular system + boost | 25.2/36.0 | VMAT | No | Alive, NED | Pan-hypo and diabetes insipidus |
| 3 | 18 | M | Germinoma | Negative | Negative | Negative | Pineal | Complete | No | Ventricular system + boost | 25.2/36.0 | VMAT | No | Alive, NED | No |
| 4 | 11 | M | Germinoma | Negative | BHCG | Negative | Pineal and suprasellar | Incomplete | No | Ventricular system + boost | 18.0/30.6 | VMAT | No | Alive, NED | Diabetes insipidus |
| 5 | 12 | M | Germinoma | Negative | BHCG | Negative | suprasellar | Complete | No | Ventricular system + boost | 25.2/36.0 | VMAT | No | Alive, NED | Diabetes insipidus |
| 6 | 15 | M | Germinoma | Negative | Negative | Negative | Pineal and suprasellar | Complete | No | Ventricular system + boost | 25.2/36.0 | VMAT | No | Alive, NED | Pan-hypo and amaurosis at diagnosis |
| 7 | 14 | M | NGGCT | AFP | AFP | Negative | Pineal | Incomplete | NE (post-CT) | Ventricular system + boost | 30.6/54.0 | VMAT | No | Alive, NED | No |
| 8 | 16 | M | Germinoma | Negative | Negative | Negative | Pineal | Complete | No | Ventricular system + boost | 18.0/30.6 | VMAT | No | Alive, NED | No |
| 9 | 13 | M | Germinoma | Negative | Negative | Negative | Suprasellar to III ventricle | Incomplete | NE (post-CT) | Ventricular system + boost | 25.2/45.0 | IMRT | No | Alive, NED | Pan-hypo |
| 10 | 12 | F | Germinoma | Negative | Negative | Negative | Sellar and suprasellar | Complete | NE (pre-CT) | Ventricular system + boost | 30.6/45.0 | IMRT | No | Alive, NED | Pan-hypo and dyslipidemia |
| 11 | 6 | M | Germinoma | Negative | Negative | Negative | Suprasellar and pineal | Incomplete | No | Ventricular system + boost | 23.4/36.0 | VMAT | No | Alive, NED | Pan-hypo |
| 12 | 11 | M | Germinoma | Negative | Negative | Negative | Pineal | Complete | NE (pre-CT) | Ventricular system + boost | 25.2/36.0 | VMAT | No | Alive, NED | Pan-hypo |
| 13 | 8 | M | Germinoma | Negative | Negative | Negative | Pineal | Complete | NE (pre-CT) | Ventricular system + boost | 22.0/36.4 | VMAT | No | Alive, NED | No |
| 14 | 7 | M | Germinoma | Negative | Negative | Negative | Suprasellar | Complete | No | Ventricular system + boost | 24.0/30.0 | IMRT | No | Alive, NED | No |
| 15 | 13 | M | NGGCT | Negative | Negative | Negative | Pineal | Complete | Total (pre-CT) | Ventricular system + boost | 30.6/54.0 | IMRT | No | Alive, NED | No |
| 16 | 12 | F | Germinoma | Negative | Negative | Negative | Suprasellar | Complete | No | Ventricular system | 30.0 | IMRT | No | Alive, NED | Pan-hypo and low stature |
| 17 | 11 | M | NGGCT | Negative | AFP/BHCG | Negative | Pituitary/pineal stalk | Complete | No | Ventricular system + boost | 30.6/50.4 | VMAT | Yes | Alive, NED | No |
| 18 | 17 | M | Germinoma | Negative | Negative | Negative | Suprasellar | Complete | Partial (pre-CT) | Ventricular system | 25.2 | IMRT | No | Alive, NED | Pan-hypo and auditory deficit |
| 19 | 8 | F | NGGCT | AFP/BHCG | AFP/BHCG | Negative | Suprasellar | Complete | No | Ventricular system + boost | 25.2/36.0 | VMAT | Yes | Dead | Pan-hypo |
| 20 | 14 | M | Germinoma | Negative | Negative | Negative | Suprasellar and pineal | Incomplete | No | Ventricular system + boost | 25.2/45.0 | IMRT | No | Alive, NED | Diabetes insipidus |

NGGCT = non-germinomatous germ cell tumors; CSF marker = cerebrospinal fluid tumor marker; CSF cytology= cytological dissemination in the cerebrospinal fluid; CT = chemotherapy; NE = not evaluated; RT Volumes = volumes receiving radiation therapy; VMAT = volumetric intensity-modulated arc radiotherapy; IMRT = intensity-modulated radiation therapy; NED = disease-free survival; pan-hypo = pan-hypopituitarism; AFP = alpha-fetoprotein; BHCG = beta human chorionic gonadotropin.
